# mGem: *Faecalibacterium*, an important protector of gut health

**DOI:** 10.1128/mbio.02776-25

**Published:** 2025-11-25

**Authors:** Carlos Sabater, Xenia Vázquez, Sylvia H. Duncan

**Affiliations:** 1 Rowett Institute, University of Aberdeen 1019, Aberdeen, Scotland, United Kingdom; 2 Group of Functionality and Ecology of Beneficial Microorganisms (MicroHealth), Dairy Research Institute of Asturias (IPLA-CSIC) 111627, Oviedo, Asturias, Spain; 3 Health Research Institute of Asturias (ISPA) 594910 , Oviedo, Asturias, Spain; The Ohio State University, Columbus, Ohio, USA

**Keywords:** *Faecalibacterium*, probiotics, gut health, anaerobes, pathogen inhibiton

## Abstract

*Faecalibacterium* is among the most abundant bacterial genera in the healthy human colon, comprising approximately 10–15% of the total gut microbiota. Species within this genus ferment complex carbohydrates, including pectin, to produce butyrate, a short-chain fatty acid with anti-inflammatory and anti-carcinogenic properties. Butyrate is the primary energy source for colonocytes and in *Faecalibacterium* is synthesized via the butyryl-CoA:acetate CoA transferase pathway. Reduced levels of *Faecalibacterium* are often associated with increased abundance of *Escherichia coli* and may be linked to early-onset colorectal cancer. Here, genomic analysis of *Faecalibacterium* strains revealed that several lack antibiotic resistance genes, suggesting a favorable safety profile. Additional genome mining revealed multiple biosynthetic gene clusters (BGCs) involved in the synthesis of secondary metabolites, including ranthipeptides, which may exhibit antimicrobial activity. Understanding the functional roles of these BGCs, particularly their potential to inhibit *E. coli*, is critical for advancing microbiome-based therapies. Moreover, developing effective delivery strategies to maintain *Faecalibacterium* populations in the colon is essential for promoting gut health and preventing disease.

## PERSPECTIVE

## *FAECALIBACTERIUM*: A KEYSTONE GENUS

The adult human colon hosts a complex and dense ecosystem of bacteria, fungi, and viruses, and the former are comprised of many hundreds of different bacterial species (1). Despite considerable interindividual variation in microbial composition, the microbiota tends to be quite stable in most adults (2) but can show changes along the life course. In particular, the frail elderly tend to have a decline in species diversity (3).

The composition of each person’s microbiota can, however, be driven by a number of factors, including diet (4, 5), colonic pH, oxygen gradients in the colon, and bile salt levels (6–10). These microbes can play a key role in health and disease, including gut health and also a range of other health factors, such as gut-brain axis signaling (11, 12).

**Fig 1:**
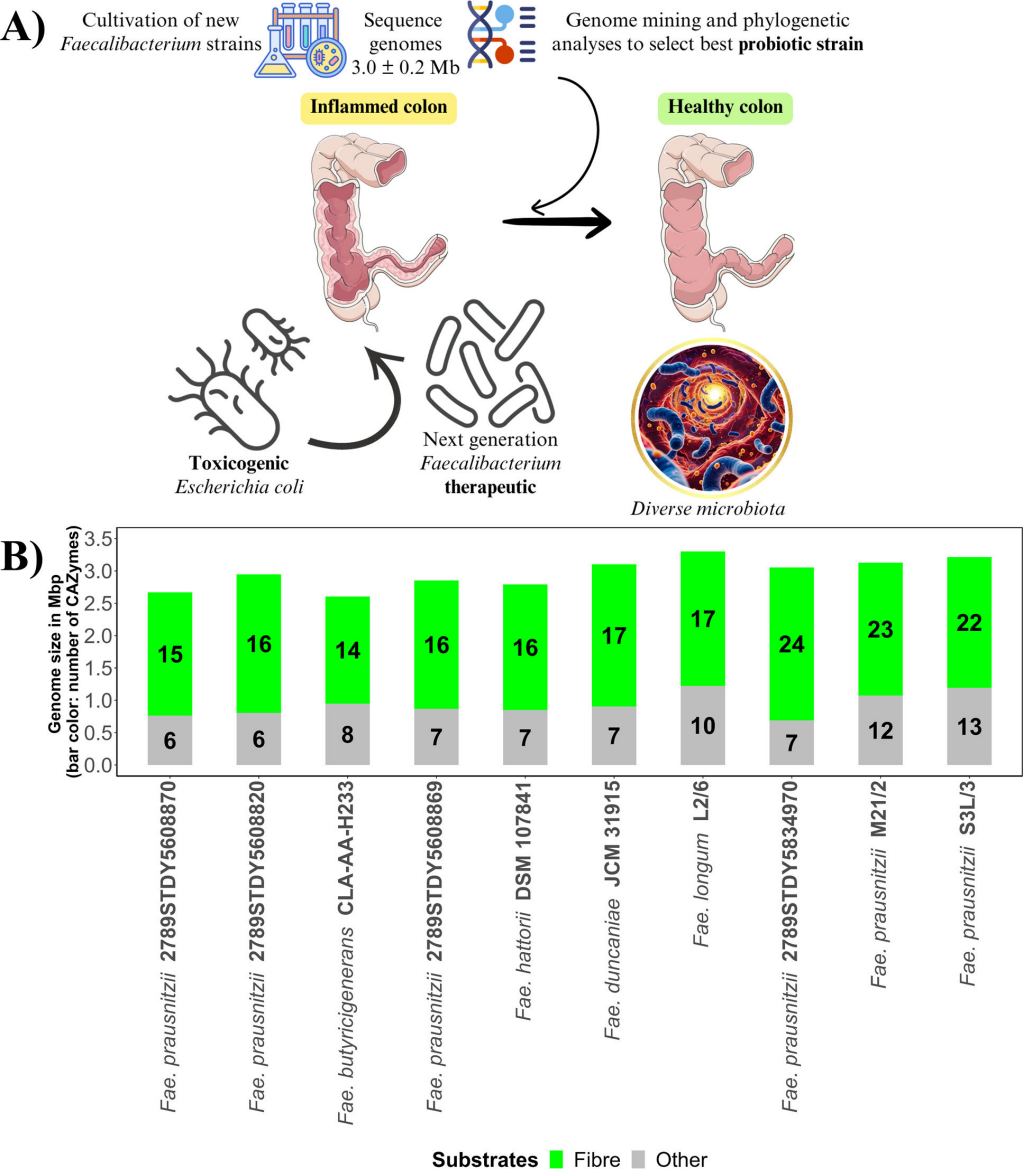
(**A**) Use of *Faecalibacterium* strains as next-generation probiotics and their role as anti-inflammatory gut microbes. This figure has been designed using resources from flaticon.com (free icons “Bacteria” by cube29, “Wand free” by Sir.Vector, “Experiment” by Paul J., and “Dna Strand” by Freepik) and bioicons.com (“healthy-colon-3d” and “crohns-disease” icon by Servier, licensed under CC-BY 3.0). (**B**) Total number of carbohydrate-active enzymes (CAZymes) acting on fiber (defined as the sum of pectin, xylan/arabinoxylan, mannan, glucan/xyloglucan, fructan, lignocellulosic materials, resistant starch, and malto-oligosaccharides) and other carbohydrate substrates (“Other”) determined in the genome sequences of currently recognized human gut *Faecalibacterium* strains. CAZymes were annotated using “run_dbcan” software (19), which maps the samples against the CAZy database (http://www.cazy.org/ last accessed: 16 June 2025). Genome sequences were retrieved from the National Center for Biotechnology Information (NCBI) repository.

In most healthy adults, the single most abundant genus is *Faecalibacterium*, which has been detected in over 85% of human intestinal samples (13), routinely making up around 5–10% of the total microbiota and can be higher in certain individuals (1, 14). In disease states, its abundance can often be much lower (15). The *Faecalibacterium* genus, first described by Duncan et al. (16), belongs to the Bacillota (previously Firmicutes) phylum (15) with a genome size of approximately 3.0 ± 0.2 Mb (Fig. 1A). Species within the *Faecalibacterium* genus include the human-derived species *F. prausnitzii*, *F. butyricigenerans*, *F. duncaniae* (17), *F. longum* (18), and *F. hattorii,* while *F. gallinarum* has been reported in chickens (15).

*Faecalibacterium* strains are non-spore forming and non-motile rod-shaped cells (16, 20). Moreover, it is a strict anaerobe (16), surviving for less than two minutes following exposure to air (21). Paradoxically, it can, however, be found in sites near the colon wall, where there is diffusion of oxygen and survival could be due to a mechanism that involves extracellular electron shuttling (22). *Faecalibacterium* strains that have been tested have revealed a degree of sensitivity to bile salts (9). Moreover, certain *Faecalbacterium* strains showed auxotrophy for vitamins and amino acids (23), suggesting reliance on other microbes for key vitamin and growth factor requirements. Given these findings, it is remarkable that *Faecalibacterium* can survive and maintain a dominant presence in the human colon.

Dietary substrates fermented by *Faecalibacterium* strains, determined using *in vitro* analyses and genome mining, include pectin (24), xylan, and mannan (25) derivatives (see Fig. 1B). *Faecalibacterium* strains showing a wide range of carbohydrate-active enzymes (CAZymes) acting on fiber include *F. prausnitzii* strains 2789STDY5834970, M21/2, and S3L-3 (*n* = 22–24) (Fig. 1B). Fermentation of simple sugars, such as glucose, and other dietary residues that escape digestion by host enzymes results in the formation of butyrate as a major fermentation end product, with minor amounts of other acids, including formate and lactate (16).

## ROLE OF *FAECALIBACTERIUM* IN HUMAN HEALTH

*Faecalibacterium* is likely to be a major contributor to human health, in part due to its fermentative abilities, which include butyrate formation. As most colonic butyrate producers, *Faecalibacterium* employs the butyryl CoA:acetate CoA transferase route for butyrate formation. Butyrate is the main energy source for colonocytes and has anti-inflammatory and anti-carcinogenic properties. Moreover, butyrate has a role in host protection via epigenetic changes during the differentiation of monocytes to macrophages. Butyrate induces the latter to upregulate antimicrobial proteins in the gut, such as calprotectin, which plays a crucial role in inflammatory responses and is used as a biomarker to detect intestinal inflammation (26). *Faecalibacterium* species and other dominant butyrate producers, such as *Eubacterium rectale* and *Roseburia* species (27), also act as anti-inflammatory gut microbes (28). Further, there is a correlation between high populations of *F. prausnitzii*, low IL-12 abundance, and higher IL-10 production (24, 29). A protein (referred to as the MAM protein), which is produced by *F. prausnitzii*, has been linked to its anti-inflammatory effects (30).

With regard to disease conditions, lower-than-usual levels of *F. prausnitzii* have been associated with inflammatory bowel disease (IBD) and Crohn’s disease (29) (Fig. 1A). Intestinal disorders, such as Crohn’s disease, found reduced levels of *F. prausnitzii* in both fecal and mucosal samples (31). The lower abundance of these bacteria is not only associated with the risk of developing IBD, but also with the chance of relapsing after successful treatment. Individuals with lower abundance of *F. prausnitzii* were six times more likely to relapse in the future (29, 32). Moreover, lower levels of *Faecalibacterium* generally correlate with elevated levels of *Escherichia coli* (7). In particular, exposure to colibactin-producing *E. coli,* particularly in early life, may be a contributing factor in the increasing early onset of colorectal cancer (32). A reduction in *Faecalibacterium* abundance and its ability to generate secondary metabolites is likely to benefit the survival of pathogenic species.

**Fig 2:**
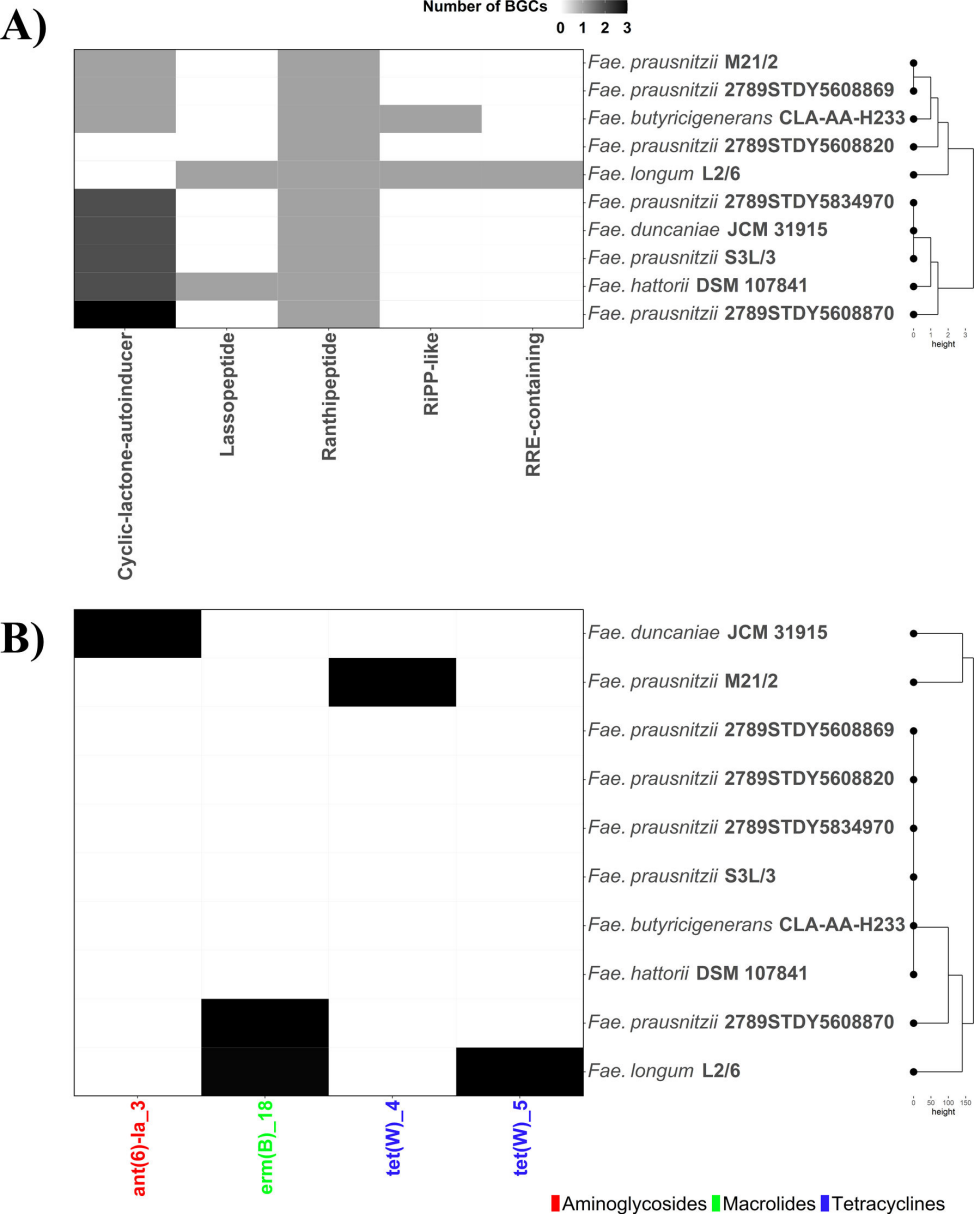
(**A**) Identification of secondary metabolite biosynthesis gene clusters (BGCs) in the genome sequences of currently recognized human gut *Faecalibacterium* strains using antiSMASH software (37). RiPP-like: other unspecified ribosomally synthesized and post-translationally modified peptide product (RiPP). RRE-containing: RiPP precursor recognition element (RRE)-element containing cluster. (**B**) Identification of antimicrobial resistance genes (ARGs) in the genome sequences of currently recognized human gut *Faecalibacterium* strains using abricate software and ResFinder database (38). Genome sequences were retrieved from the National Center for Biotechnology Information (NCBI) repository.

Several biosynthesis gene clusters (BGCs) involved in the synthesis of secondary metabolites have been identified in the genome sequences of different *Faecalibacterium* strains (Fig. 2A). Specifically, *Faecalibacterium* strains have ranthipeptide BGCs, while lassopeptide BGCs were characteristic of the *F. prausnitzii* strain L2/6. Ranthipeptides are a subclass of ribosomally synthesized and post-translationally modified peptides that share certain similarities to lanthipeptides. The latter have been shown to disrupt bacterial membranes and inhibit bacterial cell wall synthesis in Gram-negative bacteria including *E. coli* (33). Ranthipeptides have also been reported to play an important role in quorum sensing mechanisms (34), while lassopeptides show biological effects against *E. coli*, *Salmonella*, *Klebsiella*, and *Shigella* species (35, 36).

*F. prausnitzii* therefore shows excellent promise as a bacterium for supporting good health and improving gut barrier function (39, 40) (Fig. 1A). In addition, this bacterium improves the gut barrier by improving the permeability and the expression of tightly bound proteins, including occludin, and both of these increase the tight junctions between cells, thereby strengthening gut barrier function and alleviating inflammation (41).

Supporting populations of *Faecalibacterium* in the colon may require a number of different approaches, such as interactions with other bacterial species to create a suitable environment, including reducing the redox potential and altering the composition of nutrients (42). Studies show that *F. prausnitzii* interacts with other bacteria, which influences its butyrate production and survival. As an example, co-culturing *F. prausnitzii* and bifidobacteria enhanced butyrate production by *F. prausnitzii* (43) due to the supply of acetate and other nutrients. The supply of acetate helps fuel the butyryl CoA:acetate CoA pathway for butyrate formation, and certain bacteria may also supply other key growth factors, such as vitamins. *F. prausnitzii* is auxotrophic for most vitamins, including biotin, folate, and thiamine, as well as the amino acid tryptophan (23), reveals its likely reliance on other vitamin- and nutrient-synthesizing gut microbes. Cross-feeding is therefore likely to be essential for *Faecalibacterium* strains to maintain their dominance in the human colon.

Where *Faecalibacterium* is in low abundance, it is becoming increasingly important to consider how best to ameliorate this and how to deliver this bacterium to the colon. One option may be fecal microbiota transplantation (FMT), which is the transfer of fecal microbiota from a healthy donor into another individual to promote health. This method has been used with some success to treat a few health conditions, including bowel disorders and infections, particularly those caused by *Clostridioides difficile* (44, 45); however, it can have side effects, such as bacteremia (46). Given the reported relative success of FMT and the likely prevalence of *Faecalibacterium* in stool samples from healthy donors, there is considerable credible support for the safety and efficacy of developing *Faecalibacterium* as a single strain or defined microbial consortia for human use. Using single strains or defined bacterial consortia allows for a much more detailed analysis of the strains of interest, including their antibiotic resistance profiles, providing much more confidence in the use of next-generation probiotic products (47).

The use of well-characterized strains is therefore a much safer, more acceptable, and targeted approach compared to FMT to treat gut health conditions. The sensitivity of *Faecalibacterium* to oxygen and how best to deliver this anaerobe, either alone or in combination with other strains, to the intestine, however, requires further exploration. When considering the use of *Faecalibacterium* strains as a next-generation probiotic or biotherapeutic (Fig. 1A), each strain needs to be thoroughly assessed for safety and other factors, such as carriage of antibiotic resistance genes (ARGs), given the level of antibiotic resistance in intestinal bacteria. Aminoglycoside (strain JCM 31915), macrolide (strains L2/6 and 2789STDY5608870), and tetracycline (strains L2/6 and M21/2) resistance genes have been annotated in the genome sequence of *Faecalibacterium* strains (Fig. 2B). However, no multidrug-resistant *Faecalibacterium* strains have been identified. Moreover, several strains had no evidence of ARGs (Fig. 2B). It is important to consider that the strains have all factors required for growth and may therefore require adding vitamins and/or another strain (co-culture) such as *Bifidobacterium adolescentis* which could provide growth factors such as acetate to fuel the butyrate pathway (43). Given that *Faecalibacterium* does not sporulate and is sensitive to air, if using a microencapsulation approach, these factors also need to be taken into consideration, and there are methods currently used for anaerobes (48). With the increasing number of *Faecalibacterium* strains that have been isolated and genome-sequenced, it is timely to carry out a deep dive into these strains to determine how many different species exist and to compare important traits across strains, in order to optimize the choice of strain(s) for development as next-generation probiotics to best promote and potentially identify prebiotic approaches for promoting gut health. Moreover, despite growing interest in microbial metabolites, our understanding of the capacity of the human gut microbiota to produce secondary metabolites and inhibit pathogens remains limited. Uncovering this microbial “dark matter” is essential for advancing therapeutic strategies and deepening our understanding of host–microbe interactions.
